# Reduced B Lymphoid Kinase (Blk) Expression Enhances Proinflammatory Cytokine Production and Induces Nephrosis in C57BL/6-*lpr/lpr* Mice

**DOI:** 10.1371/journal.pone.0092054

**Published:** 2014-03-17

**Authors:** Elizabeth M. Samuelson, Renee M. Laird, Amber M. Papillion, Arthur H. Tatum, Michael F. Princiotta, Sandra M. Hayes

**Affiliations:** 1 Department of Microbiology and Immunology, State University of New York Upstate Medical University, Syracuse, New York, United States of America; 2 Department of Pathology, State University of New York Upstate Medical University, Syracuse, New York, United States of America; INSERM-Université Paris-Sud, France

## Abstract

*BLK*, which encodes B lymphoid kinase, was recently identified in genome wide association studies as a susceptibility gene for systemic lupus erythematosus (SLE), and risk alleles mapping to the *BLK* locus result in reduced gene expression. To determine whether *BLK* is indeed a *bona fide* susceptibility gene, we developed an experimental mouse model, namely the Blk^+/−^.*lpr/lpr* (Blk^+/−^.*lpr*) mouse, in which *Blk* expression levels are reduced to levels comparable to those in individuals carrying a risk allele. Here, we report that Blk is expressed not only in B cells, but also in IL-17-producing γδ and DN αβ T cells and in plasmacytoid dendritic cells (pDCs). Moreover, we found that solely reducing Blk expression in C57BL/6-*lpr*/*lpr* mice enhanced proinflammatory cytokine production and accelerated the onset of lymphoproliferation, proteinuria, and kidney disease. Together, these findings suggest that *BLK* risk alleles confer susceptibility to SLE through the dysregulation of a proinflammatory cytokine network.

## Introduction

Systemic lupus erythematosus (SLE) is a chronic multisystem autoimmune disorder that afflicts more than 1.5 million Americans. There is strong evidence for a genetic basis to this disease, and many candidate genes, which predispose an individual to SLE, have been identified from studies in patients with SLE and in mouse models of lupus [1]–[3]. With recent advances, however, such as the completion of the Human Genome Project and the International HapMap Project, it is now possible to perform genome-wide association studies to identify additional susceptibility genes in humans. Indeed, several groups, using this experimental approach, have identified and confirmed over 25 new susceptibility genes in SLE patients of different ethnicity and race [4]–[10]. Notably, many of these new susceptibility genes are not among those known to be associated with autoimmune disease; therefore, follow-up studies are necessary to determine the mechanisms by which they promote development of SLE.

One of the newly identified susceptibility genes is *BLK*, which encodes B lymphoid kinase (Blk). Multiple single nucleotide polymorphisms (SNPs) in the *BLK* locus, mapping primarily to the promoter and first intron, are associated with disease risk [4]–[10]. A handful of these SNPs have been studied in more depth to determine how the specific nucleotide change affects *BLK* expression. All studies to date report a 25 to 70% reduction in *BLK* expression depending on whether individuals are heterozygous or homozygous for the risk allele [5], [11]–[13]. These findings suggest that the genetic variants in the *BLK* locus predispose an individual to SLE by reducing Blk expression.

Blk was first described over two decades ago as a B cell-specific member of the Src family of tyrosine kinases (SFKs) [14]. Even though early reports demonstrated functional redundancy among Blk, Lyn, and Fyn in B cell development and activation [15], [16], a recent report has revealed a requirement for wild-type levels of Blk in the development and function of marginal zone (MZ) B cells [17], a mature splenic B cell subset involved in both microbial immunity and autoimmunity [18]. In both Blk^+/−^ and Blk^−/−^ mice, MZ B cells are fewer in number but exhibit augmented *in vitro* and *in vivo* responses to BCR stimulation in comparison to Blk^+/+^ mice [17]. With age, this B cell hyperactivity leads to autoimmunity, as evidenced by the display of multiple autoimmune phenotypes in 6-month-old Blk^+/−^ mice, including increased numbers of MZ and B1 B cells, detection of B cells with an activated surface phenotype, and production of a low but significant level of serum anti-nuclear antibodies (ANAs) [17]. Given the well-documented role of B cells in autoimmune disease development and pathogenesis [19], these data suggest that *BLK* risk alleles promote development of SLE by altering BCR signaling responses and, by extension, B cell development, function, and tolerance.

It is important to note that Blk is also expressed outside of the B cell lineage, in both immune and non-immune cells. In humans, Blk is expressed in unfractionated thymocytes, γδ T cells, plasmacytoid dendritic cells (pDCs), and pancreatic β cells [13], [20]–[22], while in mice, it is expressed in bone marrow progenitor cells, immature CD4^−^ CD8^−^ (double negative; DN) thymocytes, γδ thymocytes, IL-17-producing γδ T (γδ-17) cells, and pancreatic β cells [22], [23]. More importantly, analysis of Blk-deficient mice has revealed a requirement for Blk not only in early T cell development but also in the development and function of γδ-17 cells [23]. Therefore, because Blk is expressed in a diverse array of immune cells, it is conceivable that reducing its expression could have wide-ranging effects on immune cell development, activation, and effector function.

To determine whether and how reduced Blk expression levels contribute to autoimmune disease development and pathogenesis, we established an experimental mouse model in which *Blk* transcript and Blk protein levels are reduced by approximately 45% [17], which is within the range reported for individuals carrying a *BLK* risk allele [5], [11]–[13]. In addition, the mouse model carries the *lpr* mutation in Fas, which not only results in severely reduced Fas expression but also accelerates the development of disease when introgressed on an autoimmune susceptible background [24], [25]. We present here that, in addition to B cells and γδ-17 cells, Blk is expressed in murine pDCs and in IL-17-producing DN αβ T (DN-17) cells. Furthermore, we found that solely reducing Blk expression in B6.*lpr* mice enhanced proinflammatory cytokine production by both Blk-positive and –negative immune cells and accelerated the onset of lymphoproliferation, proteinuria, and kidney disease. These findings demonstrate that *BLK* is indeed a *bona fide* susceptibility gene and suggest that *BLK* risk alleles promote autoimmune disease development through the dysregulation of a proinflammatory cytokine network.

## Materials and Methods

### Ethics statement

All research involving animals has been conducted according to the relevant national and international guidelines with respect to husbandry, experimentation and welfare. Mouse protocols were approved by the State University of New York (SUNY) Upstate Medical University Committee on the Humane Use of Animals (CHUA protocol numbers 262 and 368).

### Mice

C57BL/6J (B6) and B6.MRL-*Fas^lpr^*/J (B6.*lpr*) mice were purchased from The Jackson Laboratory (Bar Harbor, ME, USA). B6.*Blk^tm1^* (Blk^−/−^) mice [15] were provided by A. Tarakhovsky (Rockefeller University) and V_H_3H9 site directed-transgenic (3H9 Tg) mice [26] on the B6 background were provided by M. G. Weigert (University of Chicago). All mice used in this study were bred and maintained in a barrier facility in the Department of Laboratory Animal Resources at SUNY Upstate Medical University in accordance with the specifications of the Association for Assessment and Accreditation of Laboratory Animal Care.

### Flow cytometric analysis

Flow cytometric analysis for surface antigen expression was performed by pre-incubating cells with the anti-CD16/CD32 antibody for at least 10 minutes to block non-specific binding of immunoglobulins to Fc receptors, followed by staining with fluorochrome-conjugated mAbs against various surface antigens. Intracellular staining for Blk, RORγt, T-bet, and Foxp3 was performed using the Foxp3/Transcription factor staining buffer set (eBioscience, San Diego, CA, USA) according to the manufacturer's instructions. Intracellular staining for IL-6, TNFα, IL-17A, IFNγ, and IL-10 was performed by first fixing cells in a final concentration of 1.5% formaldehyde for 10 minutes at 37°C. Fixed cells were stained for surface antigens, permeabilized with Perm/Wash Buffer (BD Pharmingen, San Jose, CA, USA) for 20 minutes at 4°C, and then stained with antibodies against the appropriate cytokines. For all experiments, 0.5 to 1 × 10^6^ cells were acquired on a BD LSRFortessa using FACSDiva software (BD Immunocytometry Systems, San Jose, CA, USA). Data analysis was performed using FlowJo software (Tree Star, Inc., Ashland, OR, USA). Dead cells were excluded from analysis based on forward and side scatter profiles.

Antibodies used for flow cytometric analysis included anti-B220 (RA3-6B2), anti-CCR6 (29-2L17), anti-CD1d (1B1), anti-CD3 (145-2C11), anti-CD4 (RM4-5), anti-CD5 (53-7.3), anti-CD8α (53-6.7), anti-CD11b (M1/70), anti-CD11c (N418), anti-CD19 (6D5), anti-CD21 (7E9), anti-CD22.2 (Cy34.1), anti-CD23 (B3B4), anti-CD25 (PC61), anti-CD44 (IM7), anti-CD62L (MEL-14), anti-CD86 (GL-1), anti-CD93 (AA4.1), anti-CD138 (281-2), anti-CD317 (927), anti-NK1.1 (PK136), anti-CXCR5 (L138D7), anti-F4/80 (BM8), anti-IA^b^ (AF6-120.1), anti-ICOS (C398.4A), anti-ICOSL (HK5.3), anti-BAFF-R (7H22-E16), anti-IgM (RMM-1), anti-IgM^a^ (MA-69), anti-IgM^b^ (AF6-78), anti-IgD (11-26c.2a), anti-Igλ1 (R11-153), anti-TCRγδ (UC7-13D5), anti-TCRβ (H57-597), which were purchased from BD Biosciences, eBioscience or BioLegend (San Diego, CA, USA). Antibodies used for intracellular staining were anti-Foxp3 (FJK-16s; eBioscience), anti-T-bet (4B10; eBioscience), anti-IL-6 (MP5-20F3; eBioscience), anti-IL-10 (JES5-16E3; eBioscience), anti-IL-17A (TC11-18H10.1; BioLegend), anti-IFNγ (XMG1.2; BD Biosciences), anti-TNFα (MP6-XT22; BioLegend), anti-RORγt (B2D; eBioscience), anti-Blk (Cell Signaling Technology, Danvers, MA) and FITC-donkey anti-rabbit IgG (Invitrogen, Grand Island, NY).

### ELISAs

Cytokine, IgM, IgG, and autoantibody levels in supernatants or sera were determined with ELISA kits purchased from eBioscience, BioLegend, R and D systems (Minneapolis, MN, USA), or Alpha Diagnostics (San Antonio, TX, USA).

### *In vitro* stimulation of immune cell subsets

To assess pDC function, pDCs from 2-month-old B6.*lpr* or Blk^+/−^.*lpr* spleens were purified by negative selection using the Mouse Plasmacytoid Dendritic Cell Isolation Kit II (Miltenyi, Auburn, CA, USA) and then stimulated with Type C CpG ODN 2395 (1 μg/ml; Invivogen, San Diego, CA, USA) for 24 hours at 37°C. Supernatants were collected and assayed for IFNα by ELISA.

To polarize naïve CD4^+^ T cells towards the Th17 and Tfh effector lineages, naïve CD4^+^ T cells were purified from the spleens and peripheral lymph nodes (pLNs) of B6 mice by negative selection using magnetic bead separation as previously described [23]. Cells were stimulated with 5 μg/ml of immobilized anti-CD3 antibody and 1 μg/ml of soluble anti-CD28 antibody in the presence of 1 ng/ml anti-IFNγ antibody, 1 ng/ml anti-IL-4 antibody, and either IL-6 (100 ng/ml; Miltenyi) for Tfh polarization or IL-6 (100 ng/ml) plus TGFβ (1 ng/ml; Miltenyi) for Th17 polarization. After 3 days at 37°C, cells were harvested and their Blk expression levels were analyzed by flow cytometry.

For all other *in vitro* stimulation assays, 3 × 10^6^ pLN or spleen cells from 3-month-old B6, Blk^+/−^, B6.*lpr*, or Blk^+/−^.*lpr* mice were cultured for 4 hours at 37°C in the presence of brefeldin A-containing Leukocyte Activation Cocktail (BD Biosciences) to assess cytokine production by various immune cell subsets or in the presence of Cell Stimulation Cocktail (eBioscience) to measure cytokines in the supernatant.

### Measurement of Urine Protein Concentration

Protein concentrations in the urine were monitored on a weekly basis using Albustix assay strips (Siemens Healthcare Diagnostics, Tarrytown, NY, USA). Scoring was as follows: 0  =  0 mg/dl; 1  =  trace; 2  =  30 mg/dl; 3  =  100 mg/dl; 4  =  300 mg/dl; 5  =  >300 mg/dl.

### Histopathology

Kidneys, lungs, and livers were harvested, fixed in 10% neutral-buffered formalin, and then embedded in paraffin. For light microscopy, tissue samples were sectioned at 4 μm and then stained with H&E or periodic acid-Schiff (PAS). For electron microscopy, specimens were chosen from paraffin-embedded kidneys, using H&E stained sections to locate glomeruli. Specimens were reprocessed as follows: dewaxing in xylene, rehydrating in progressively diluted ethanol, post-fixing in osmium tetraoxide, dehydrating in progressively concentrated ethanol, infiltrating with propylene oxide, embedding in Epon, and then sectioning with an ultramicrotome. Glomerular damage was assessed by a renal pathologist (A.H.T.), who was blinded to the genotype of the kidney samples.

### Quantitative real-time RT-PCR analysis and RT^2^ Profiler PCR array

RNA was isolated from homogenized kidneys and splenic B cells using Qiagen's RNeasy kit, and cDNA was synthesized using Invitrogen's SuperScript First-Strand Synthesis System. The expression of genes associated with the IL-23/IL-17 axis was assessed using Qiagen's “Th17 for Autoimmunity and Inflammation” RT^2^ Profiler PCR array. For quantitative real-time PCR analysis, all primer sets, as well as the SYBR Green PCR Master Mix, were purchased from Qiagen (Valencia, CA, USA).

### Listeria Infection

Infection of mice with *Listeria monocytogenes* and subsequent analysis of T cell effector function were performed as previously described [27], except that a cocktail of IL-23 (50 ng/ml; BioLegend), IL-1 (10 ng/ml; BioLegend), and Pam_3_Cys (1 μg/ml; Invivogen) was used to elicit IL-17 production from both γδ-17 and DN-17 cells.

### Statistical analysis

Data are presented as either values for individual mice or the mean ± SEM for a group of mice. All statistical analyses were performed using GraphPad Prism software (La Jolla, CA, USA). The Student's t test was used to analyze parametric data, the Mann-Whitney *U* test was used to analyze nonparametric data, and the Spearman's rank order correlation was used to measure the strength of the relationship between TNFα serum levels and urine protein concentrations and between IgG serum levels and cytokine serum levels.

## Results

### Blk is expressed in murine B cells, γδ and DN αβ T cells with the potential to produce IL-17, and pDCs

**Figure 1:**
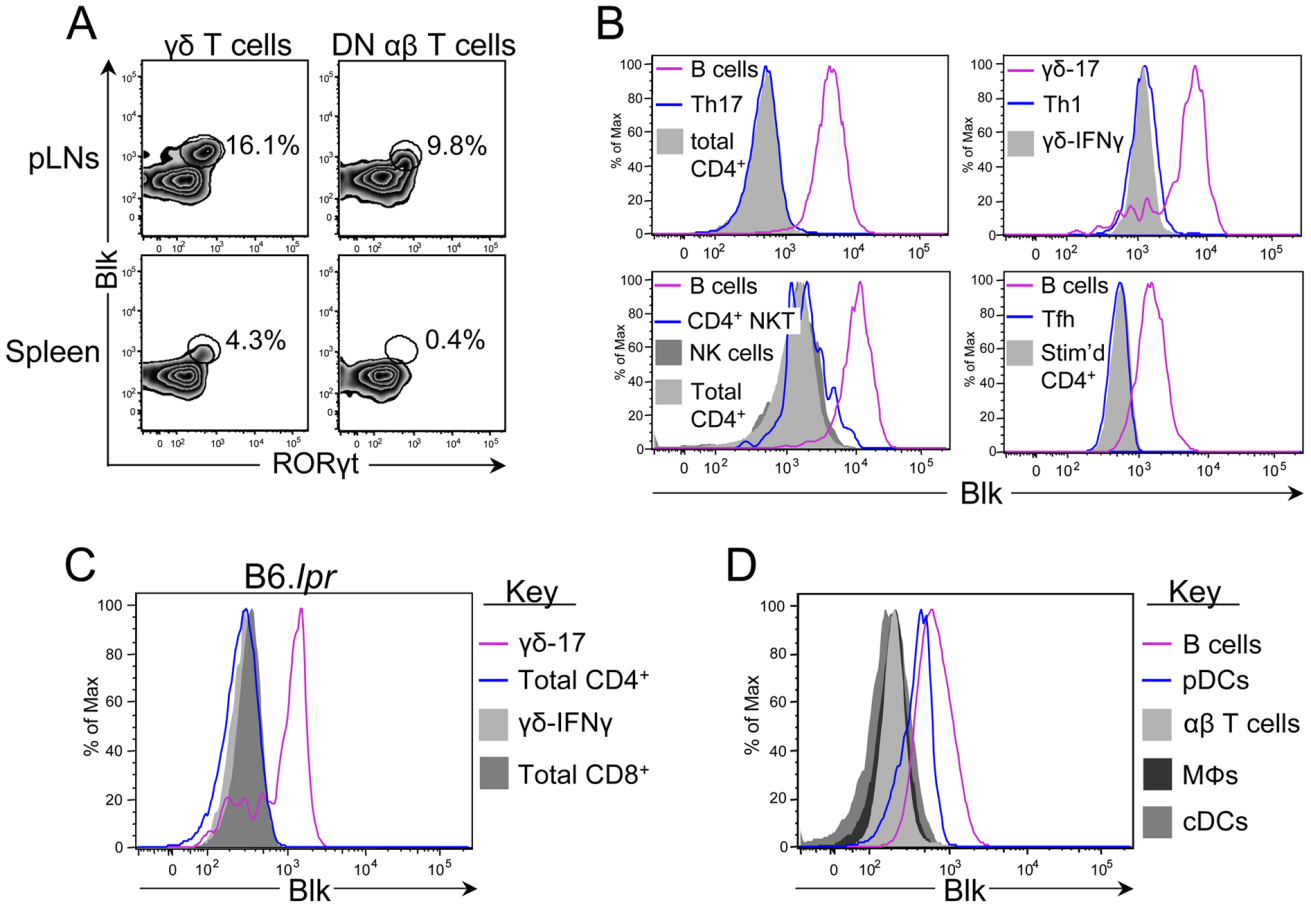
Comparison of Blk expression levels in multiple immune cell subsets. (**A**) Dot plots showing RORγt versus Blk expression in gated γδ T cells (left) and DN αβ T cells (right) from the pLNs (top) and spleen (bottom) of B6 mice. (**B**) Histograms showing Blk expression levels in various effector cell subsets. Tfh and Th17 cells were obtained by culturing TCR-stimulated naïve CD4^+^ T cells under the respective polarizing conditions. The other effector subsets were identified by surface phenotype on *ex vivo* splenocytes or pLN cells from B6 mice. Specifically, they were NK cells (NK1.1^+^ CD3^−^ CD19^−^), CD4^+^ NKT cells (CD4^+^ NK1.1^+^ TCRβ^+^ CD3^+^), and Th1 cells (CD4^+^ T-bet^+^). B cells (CD19^+^) and γδ-17 cells (CD27^−^ CCR6^+^) are shown as positive controls. CD4^+^ TCRβ^+^ CD3^+^ cells, both *ex vivo* and *in vitro* TCR-stimulated, and γδ-IFNγ cells (CD27^+^ CCR6^−^) are shown as negative controls, as previously described [23]. (**C**) Histograms showing Blk expression levels in CD4^+^ TCRβ^+^ CD3^+^ and CD8^+^ TCRβ^+^ CD3^+^ pLN cells from B6.*lpr* mice displaying lymphadenopathy. γδ-17 and γδ-IFNγ cells from B6.*lpr* mice are shown as positive and negative controls, respectively. (**D**) Histograms showing Blk expression in B cells (CD19^+^; positive staining control), pDCs (CD19^−^ CD317^+^ CD11c^+^), αβ T cells (CD3^+^; negative staining control), macrophages (MΦs; CD19^−^ CD11b^+^), and cDCs (CD19^−^ CD317^−^ CD11c^+^) from the spleens of B6 mice. For all experiments, 4 to 8 mice per genotype were used.

The first step in determining the contribution of *BLK* risk alleles to SLE development and pathogenesis is to identify which cells express this SFK. We have previously shown, using an intracellular flow cytometric assay that we developed to measure Blk expression levels, that Blk is expressed in T lineage cells, specifically immature DN thymocytes, γδ thymocytes and γδ-17 cells [23]. Here, we have extended these previous findings to include DN-17 cells (Figure 1A), which are defined by their expression of RORγt, a transcription factor required for IL-17 expression [28]. Blk, however, is not a universal marker for IL-17-producing cells, as other cell types that produce IL-17, such as T helper 17 (Th17) cells, CD4^+^ NKT cells and NK cells, did not express Blk (Figure 1B). Moreover, Blk was not detected in Th1 cells and T follicular helper (Tfh) cells (Figure 1B), both of which participate in SLE development and pathogenesis [29], [30]. Last, we did not observe Blk expression in CD4^+^ and CD8^+^ T cells from B6.*lpr* mice (Figure 1C), indicating that neither the expression of the *Fas^lpr^* mutation in T cells nor the microenvironment induced by the expression of the *Fas^lpr^* mutation resulted in abnormal expression of Blk in conventional αβ T cells. These data indicate that Blk expression in mature T lineage cells is limited to unconventional IL-17-producing T cells.

Blk, along with other components of the BCR-coupled signaling pathway, are expressed by human pDCs [21]. Notably, we found that murine pDCs also express Blk, albeit at levels lower than those observed in B cells (Figure 1D). No Blk expression, however, was detected in murine macrophages and conventional dendritic cells (cDCs) (Figure 1D). These data demonstrate that Blk is expressed in B cells, γδ-17 cells, DN-17 cells, and pDCs. Although these are functionally disparate cells, it is important to note that all of these cell types play a role in autoimmune disease development and/or pathogenesis [31]–[34].

### Development of proteinuria and nephrosis in 5-month-old Blk^+/−^.*lpr* mice

**Figure 2:**
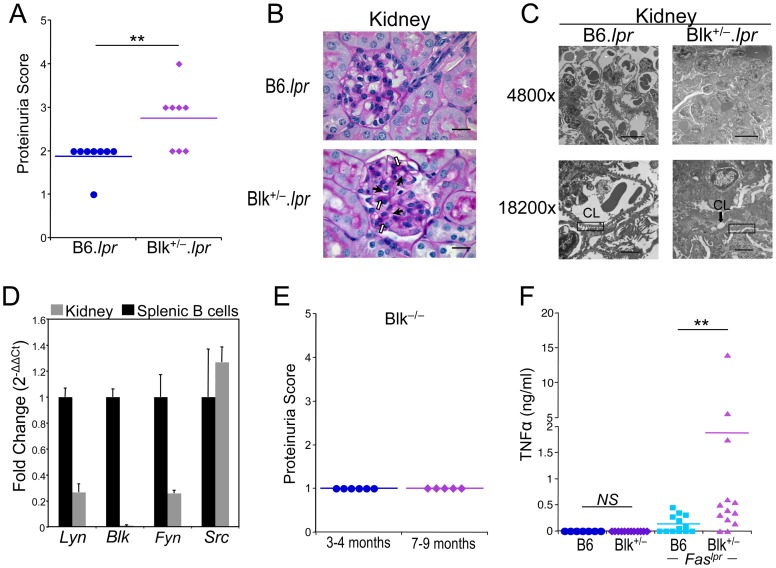
5-month-old Blk^+/−^.*lpr* mice exhibit proteinuria and nephrosis. (**A**) Comparison of proteinuria scores between 5-month-old B6.*lpr* and Blk^+/−^.*lpr* mice. 0  =  0 mg/dl; 1  =  trace; 2  =  30 mg/dl; 3  =  100 mg/dl; 4  =  300 mg/dl; 5  =  >300 mg/dl. Each symbol represents an individual mouse. (**B**) Representative light micrographs of PAS stained kidney sections from 5-month-old B6.*lpr* (n = 7) and Blk^+/−^.*lpr* (n = 8) mice at 1000× magnification. Filled arrows highlight examples of narrowed capillary lumens, while open arrows highlight examples of PAS-positive hyaline deposits. Bar  =  20 μm. (**C**) Electron micrographs of glomeruli from 5-month-old B6.*lpr* and Blk^+/−^.*lpr* mice. Line in bottom of micrographs represents 10 μm in the 4800× magnification and 2 μm in the 18200× magnification. CL, capillary lumen. Rectangular boxes highlight normal (left panel) and shortened/fused (right panel) podocyte foot processes. (**D**) Quantitative real-time RT-PCR analysis showing expression of *Src*, *Fyn*, *Lyn* but not *Blk* in B6 kidney. Data were normalized to *Gapdh* levels and are presented as fold change over purified splenic B cells (set to 1). Data represent 4 kidney samples and 4 B cell samples. (**E**) Proteinuria scores for 3- to 4-month-old and 7- to 9-month old Blk^−/−^ mice. Each symbol represents an individual mouse. (**F**) Comparison of serum TNFα levels between 5-month-old B6.*lpr* and Blk^+/−^.*lpr* mice. Each symbol represents an individual mouse. 5/13 (38.5%) of B6.*lpr* mice and 11/13 (84.6%) of Blk^+/−^.*lpr* mice have serum TNFα concentrations greater than 0.1 ng/ml. **p ≤ 0.01.

B6.*lpr* mice do not manifest severe autoimmune disease, specifically glomerulonephritis, until 9 months of age [35]. Consequently, we reasoned that if *BLK* were a *bona fide* susceptibility gene, then reducing its expression would either accelerate the onset of glomerulonephritis or increase its incidence and severity in B6.*lpr* mice. To test this, we monitored the concentration of protein in the urine, as a measure of glomerular barrier function, starting at 3 months of age. At 5 months of age, 60% of the Blk^+/−^.*lpr* mice, but none of the B6.*lpr* mice, displayed proteinuria (defined as ≥100 mg/dl) (Figure 2A). Surprisingly, by light microscopy, none of the Blk^+/−^.*lpr* mice showed mesangial proliferation or any other signs of immune complex (IC)-induced inflammation (Figure 2B). In agreement with this, we found no difference in the serum levels of ANAs between 5-month-old B6.*lpr* and Blk^+/−^.*lpr* mice (Figure S1A). We did note, however, narrowing of the capillary lumens and PAS-positive hyaline deposits in the glomeruli of Blk^+/−^.*lpr* mice but not in those of B6.*lpr* mice (Figure 2B). To explain the development of proteinuria in the absence of IC-induced inflammation, we examined the ultrastructure of the Blk^+/−^.*lpr* glomerulus by electron microscopy. In addition to the narrowed capillary lumens and hyaline deposition, we observed damage to the podocyte, a component of the glomerular filtration barrier, as evidenced by the considerable focal shortening (i.e., effacement) of their foot processes in the Blk^+/−^.*lpr* glomerulus (Figure 2C, Figure S2). Taken together, these data indicate that 5-month-old Blk^+/−^.*lpr* mice suffer from nephrosis, a kidney disease that is frequently observed in SLE patients with renal involvement [36], [37]. In fact, a recent classification, termed lupus podocytopathy, has been developed to describe SLE patients who present with nephrotic-range proteinuria and podocyte foot process effacement but no IC-induced inflammation [36], [38].

One possible explanation for the development of proteinuria in Blk^+/−^.*lpr* mice is that Blk is expressed by resident kidney cells, and that reducing its expression leads to impaired glomerular barrier function. To test this, we used quantitative RT-PCR analysis to determine whether *Blk*, along with other SFK genes, are expressed in wild-type B6 kidney. Notably, we found that cells within the kidney expressed *Src*, *Lyn*, and *Fyn* transcripts but not *Blk* transcripts (Figure 2D), which is consistent with recent reports showing that neither the *Blk* gene nor the Blk protein is expressed in total kidney, kidney cell lines and primary podocytes [39], [40]. Nonetheless, to rule out an intrinsic role for Blk in maintaining glomerular barrier function, we determined whether Blk-deficient mice develop proteinuria with age. As shown in Figure 2E, only trace amounts of protein are detected in the urine of 3- to 4-month-old as well as of 7- to 9-month-old Blk^−/−^ mice. Taken together, these data suggest that the development of proteinuria in 5-month-old Blk^+/−^.*lpr* mice is through an extrinsic mechanism.

In both humans and mice, there is a strong correlation between serum levels of TNFα and the severity of proteinuria [41], [42]. Accordingly, another possible explanation for the development of proteinuria in Blk^+/−^.*lpr* mice is that reducing Blk expression in B6.*lpr* mice promotes TNFα production. To test this, we compared TNFα serum levels in 5-month-old B6.*lpr* and Blk^+/−^.*lpr* mice and found that they were indeed significantly higher in Blk^+/−^.*lpr* mice than in B6.*lpr* mice (Figure 2F). Moreover, TNFα levels correlated with severity of proteinuria (r^2^  =  0.594; *p*  =  0.02), suggesting that elevated TNFα levels contribute to the development of proteinuria, and possibly nephrosis, in Blk^+/−^.*lpr* mice.

**Figure 3:**
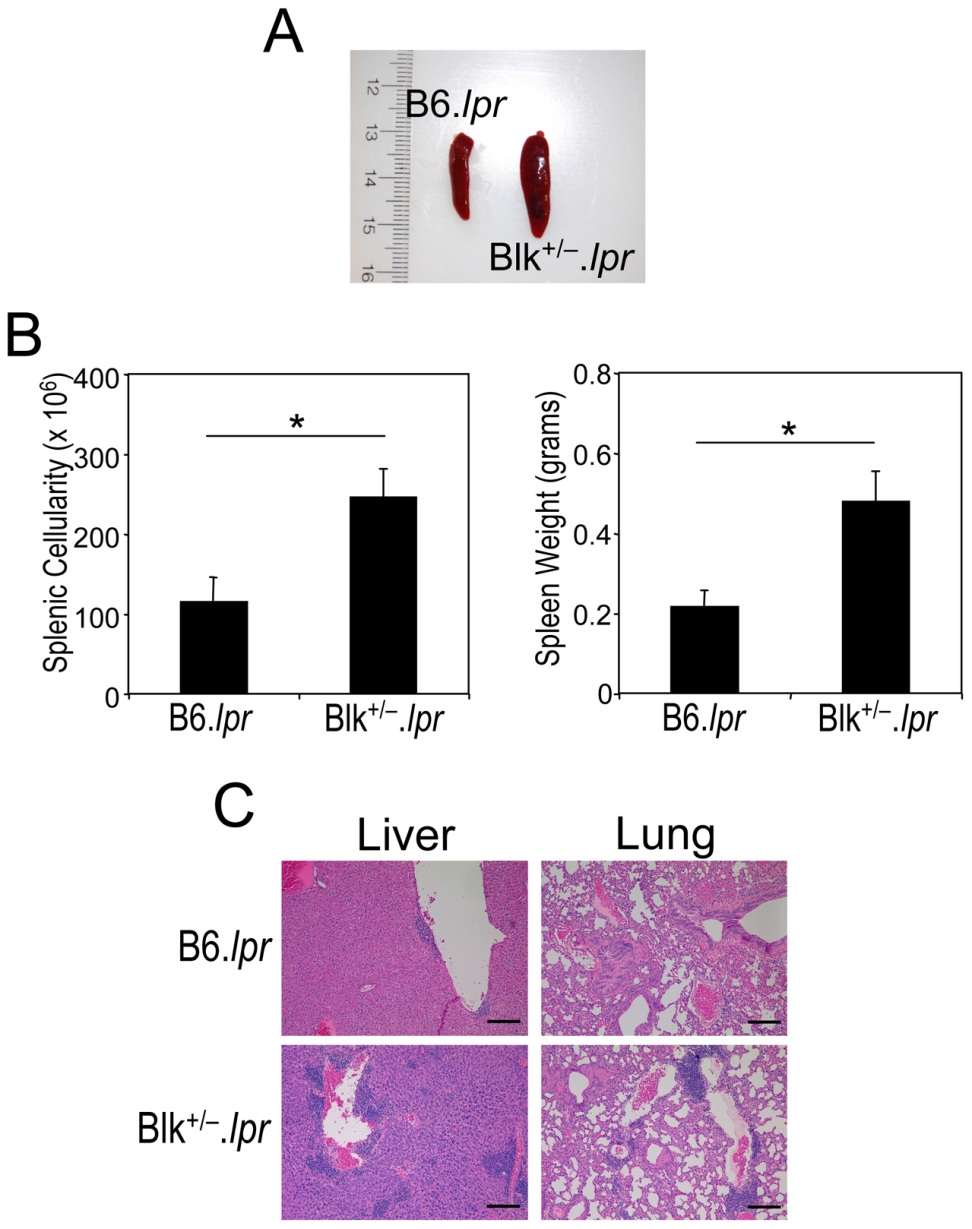
5-month-old Blk^+/−^.*lpr* mice exhibit multiple autoimmune phenotypes. (**A**) 5-month-old Blk^+/−^.*lpr* mice exhibit splenomegaly. (**B**) Comparison of splenic cellularity and weight between 5 month-old B6.*lpr* (n = 7) and Blk^+/−^.*lpr* (n = 8) mice. (**C**) Multifocal lymphocyte infiltrates are observed in the liver and lungs of 5-month-old Blk^+/−^.*lpr* mice but not age-matched B6.*lpr* mice. 100× magnification is shown. Bar  =  200 μm. *p ≤ 0.05.

Notably, other phenotypes that are associated with autoimmune disease were also observed in 5-month-old Blk^+/−^.*lpr* mice. These included splenomegaly and multifocal lymphocytic infiltration of the lung and liver (Figure 3). Collectively, these data indicate that solely reducing Blk expression levels in B6.*lpr* mice leads to early onset lymphoproliferation, proteinuria and nephrosis.

To gain insight into the mechanisms by which Blk expression levels regulate autoimmune disease development, we performed a phenotypic and functional analysis of Blk^+/−^.*lpr* mice, prior to the onset of proteinuria and nephrosis, in order to exclude any disease-related effects on immune cell function. We settled on 3 months of age because, at this age, Blk^+/−^.*lpr* mice exhibit lymphoproliferation but have minimal levels of serum autoantibodies and no serum TNFα nor proteinuria (Table 1, Figure S1B and C, data not shown).

**Table 1:** Comparison of immune cell numbers in 3-month-old B6, Blk^+/−^, B6.*lpr* and Blk^+/−^.*lpr* mice.

	B6	Blk^+/−^	*p* value	B6.*lpr*	Blk^+/−^.*lpr*	*p* value
**Spleen**	89.1 ± 4.0^a^	114.3 ± 6.8	0.002	124.7 ± 7.2	155.4 ± 9.2	0.01
Total B cells (CD19^+^)	40.8 ± 2.3	49.3 ± 3.8	0.05	62 ± 3.1	70.7 ± 4.0	0.1
Transitional (CD93^+^CD19^+^)	5.5 ± 0.5	6.1 ± 1.0	0.6	14.3 ± 1.2	15.8 ± 1.6	0.5
FO B cells (CD93^−^CD23^hi^CD21^lo^)	32 ± 2.1	36.5 ± 2.8	0.2	36.6 ± 2.2	43.2 ± 3.1	0.1
MZ B cells (CD93^−^CD23^l^°CD21^hi^)	2.9 ± 0.3	2.4 ± 0.3	0.2	4.0 ± 0.3	3.9 ± 0.4	0.9
Pre-plasmablasts (CD93^−^CD23^l^°CD21^lo^)	1.7 ± 0.2	3.1 ± 0.4	0.003	4.6 ± 0.3	5.9 ± 0.5	0.03
B1 B cells (CD5^+^IgM^+^)	0.7 ± 0.09	1.3 ± 0.1	0.002	1.5 ± 0.1	1.8 ± .09	0.1
Total T cells (CD3^+^)	33.1 ± 2.4	48.5 ± 3.4	0.0007	41.2 ± 5.5	58.0 ± 5.0	0.03
Total CD4^+^ cells	18.6 ± 1.5	26.8 ± 2.1	0.002	20.1 ± 2.4	27.8 ± 2.2	0.02
T_reg_ cells (CD4^+^Foxp3^+^)	3.4 ± 0.3	3.8 ± 0.3	0.4	5.8 ± 0.7	7.2 ± 0.5	0.1
Total CD8^+^ cells	12.8 ± 0.9	18.2 ± 1.1	0.0006	11.3 ± 1.9	13.9 ± 1.2	0.2
Total DN αβ T cells	0.9 ± 0.07	1.5 ± 0.2	0.0006	8.2 ± 1.3	14.1 ± 1.7	0.02
B220^+^ DN αβ T cells	0.2 ± 0.02	0.4 ± 0.06	0.01	6.4 ± 1.1	11.7 ± 1.6	0.02
γδ T cells	1.1 ± 0.08	1.9 ± 0.2	0.0009	1.4 ± 0.2	1.9 ± 0.2	0.07
Macrophages (CD19^−^F4/80^+^)	1.8 ± 0.2	2.5 ± 0.3	0.05	5.1 ± 0.6	5.3 ± 0.5	0.8
cDCs (CD19^−^CD11c^+^CD317^−^)	0.8 ± 0.07	1.2 ± 0.1	0.08	1.7 ± 0.1	1.8 ± 0.1	0.6
pDCs (CD19^−^CD11c^+^CD317^+^)	0.13 ± 0.01	0.22 ± 0.04	0.06	0.18 ± 0.01	0.24 ± 0.02	0.03
**Peritoneal Cavity**	1.4 ± 0.2	2.3 ± 0.5	0.08	3.6 ± 0.4	3.0 ± 0.4	0.3
B1 B cells (CD5^+^IgM^+^)	0.019 ± 0.005	0.023 ± 0.006	0.5	0.3 ± 0.007	0.4 ± 0.006	0.7
**Lymph Nodes**	26.4 ± 1.1	29.5 ± 1.5	0.1	54.0 ± 7.9	66.1 ± 9.3	0.3
Total T cells (CD3^+^)	18.8 ± 1.1	22.2 ± 1.4	0.06	33.2 ± 5.7	40.1 ± 6.3	0.4
Total CD4^+^ cells	9.5 ± 0.6	11.2 ± 0.7	0.09	9.3 ± 1.3	11.7 ± 1.8	0.3
Total CD8^+^ cells	8.7 ± 0.5	10.1 ± 0.6	0.06	10.4 ± 1.4	12.8 ± 2.2	0.4
Total DN αβ T cells	0.2 ± 0.02	0.3 ± 0.05	0.06	12.0 ± 3.3	14.0 ± 3.1	0.7
B220^+^ DN αβ T cells	0.04 ± 0.009	0.06 ± 0.01	0.2	10.7 ± 3.0	12.4 ± 2.9	0.7
γδ T cells	0.3 ± 0.02	0.4 ± 0.03	0.006	0.8 ± 0.1	1.3 ± 0.3	0.09

a Mean number of cells per tissue or subset ± SEM × 10^6^. n  =  10 to 27 mice per genotype.

### Blk is required to regulate B cell functional responses not B cell tolerance

We have previously shown that MZ B cell numbers are significantly decreased in 2-month-old Blk^+/−^ mice but, with age, MZ B cell numbers increase and, at 6 months of age, even surpass those observed in age-matched B6 mice [17]. At 3 months of age, in both Blk^+/−^ and Blk^+/−^.*lpr* mice, we found that MZ B cell numbers were equivalent to those in B6 and B6.*lpr* mice, respectively, although percentages of MZ B cells were significantly reduced in comparison to B6 and B6.*lpr* mice (Table 1, Figure S3). Except for an increase in the number of splenic B1 B cells in Blk^+/−^ mice compared to B6 mice, no differences in the numbers of mature B cell subsets were observed between B6 and Blk^+/−^ mice and between B6.*lpr* and Blk^+/−^.*lpr* mice at 3 months of age (Table 1).

**Figure 4:**
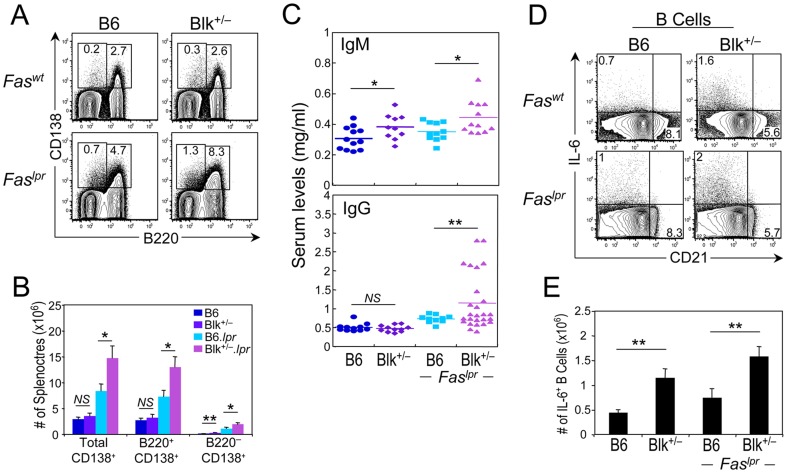
Reducing Blk expression in B6.*lpr* mice results in B cell hyperactivity. (**A**) Representative dot plots showing B220 versus CD138 expression on splenocytes from 3-month-old B6, Blk^+/−^, B6.*lpr* and Blk^+/−^.*lpr* mice. Percentages of cells that are short-lived (B220^+^ CD138^+^) and long-lived (B220^−^ CD138^+^) plasma cells are shown for each genotype. (**B**) Graph comparing number of total plasma cells, short-lived plasma cells, and long-lived plasma cells in B6 (n = 10), Blk^+/−^ (n = 10), B6.*lpr* (n  = 22) and Blk^+/−^.*lpr* (n  = 22) mice. (**C**) Comparison of the total serum levels of IgM (top) and IgG (bottom) in 3-month-old B6, Blk^+/−^, B6.*lpr* and Blk^+/−^.*lpr* mice. Each symbol represents an individual mouse. (**D**) Splenocytes from 3-month-old B6, Blk^+/−^, B6.*lpr* and Blk^+/−^.*lpr* mice were stimulated with Leukocyte Activation Cocktail for 4 hours. Dot plots showing CD21 versus IL-6 expression in gated CD19^+^ cells. Percentages of IL-6^+^ splenic B cells are shown. (**E**) Graph comparing numbers of IL-6^+^ splenic B cells in B6 (n = 4), Blk^+/−^ (n = 4), B6.*lpr* (n = 7) and Blk^+/−^.*lpr* (n = 8) mice. *p ≤ 0.05; **p ≤ 0.01.

**Figure 5:**
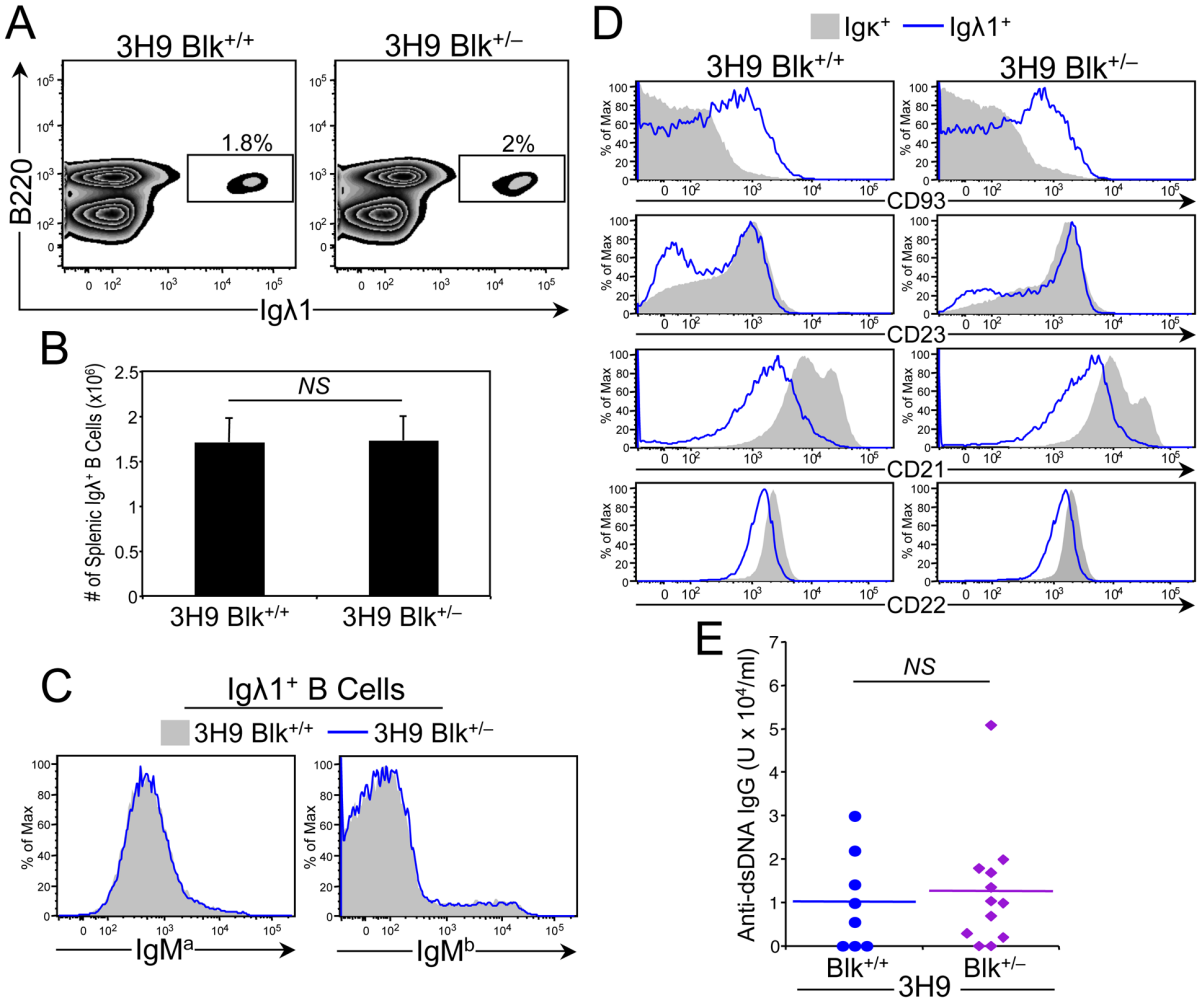
Effect of reducing Blk expression on B cell tolerance induction in 3H9 Tg mice. (**A**) Representative dot plots showing Igλ1 versus B220 expression on total splenocytes from 6-month-old 3H9 Tg Blk^+/+^ and 3H9 Tg Blk^+/−^ mice. Numbers in plots represent percentage of anti-DNA B cells (B220^+^ Igλ1^+^). (**B**) Graph comparing absolute numbers of anti-DNA B cells in the spleens of 6-month-old 3H9 Tg Blk^+/+^ (n = 7) and 3H9 Tg Blk^+/−^ (n = 8) mice. (**C**) Histograms comparing IgM^a^ (allotype of 3H9 IgH transgene) and IgM^b^ (allotype of endogenous IgH) surface levels on gated Igλ1^+^ B cells from 6-month-old 3H9 Tg Blk^+/+^ (n = 7) and 3H9 Tg Blk^+/−^ (n = 8) mice. (**D**) Histograms comparing CD93, CD23, CD21 and CD22 levels on gated Igκ^+^ (contains B cells that do not bind DNA) and Igλ1^+^ B cells from 6-month-old 3H9 Tg Blk^+/+^ (n = 7) and 3H9 Tg Blk^+/−^ (n = 8) mice. (**E**) Comparison of anti-dsDNA IgG antibodies in 6-month-old 3H9 Tg Blk^+/+^ and 3H9 Tg Blk^+/−^ mice. Each symbol represents an individual mouse.

One way B cells contribute to autoimmunity is through the production of autoantibodies [31]. We found that the serum levels of ANAs and anti-cardiolipin antibodies in 3-month-old B6.*lpr* and Blk^+/−^.*lpr* mice were low and were comparable to those in age-matched B6 and Blk^+/−^ mice (Figure S1C, data not shown). Nonetheless, we did observe significant increases in the numbers of plasma (CD138^+^) cells, both short-lived (B220^+^) and long-lived (B220^−^), and in the total serum levels of IgM and IgG in Blk^+/−^.*lpr* mice compared to B6.*lpr* mice (Figure 4A-C). Notably, we also observed more long-lived plasma cells and higher IgM serum levels in Blk^+/−^ mice than in B6 mice (Figure 4B-C). Together, these results suggested that reducing Blk expression leads to generalized B cell hyperactivity and not to a loss of B cell tolerance. To investigate this further, we generated and analyzed Blk^+/−^ mice carrying the well-described 3H9 IgH transgene, which forms an anti-DNA antibody when paired with most endogenous light chains [26]. Phenotypic analysis of 6-month-old Blk^+/+^ 3H9 Tg and Blk^+/−^ 3H9 Tg mice revealed no differences in the percentage and number of anti-DNA (Igλ1^+^ IgM^a+^) B cells, and that the vast majority of anti-DNA B cells in both genotypes had similar surface phenotypes and were arrested at the transitional (CD93^+^) B cell stage (Figure 5A-D). More importantly, equivalent levels of anti-dsDNA IgG were detected in the sera of 6-month-old Blk^+/−^ 3H9 Tg and Blk^+/+^ 3H9 Tg mice (Figure 5E). These data indicate that Blk does not act intrinsically to regulate B cell tolerance.

In addition to autoantibody production, B cells contribute to autoimmunity by secreting effector cytokines and functioning as antigen presenting cells (APCs) [31], [43]-[45]. Accordingly, we assessed the ability of B cells from 3-month-old B6, Blk^+/−^, B6.*lpr*, and Blk^+/−^.*lpr* mice to secrete proinflammatory and anti-inflammatory cytokines and to express surface antigens associated with APC function. Following a short-term PMA/ionomycin-stimulation, we found that B cells from all four genotypes produced IL-6, IFNγ, and IL-10, but not TNFα (Figure 4D, data not shown). When we quantified the number of cytokine-producing B cells, we noted ∼2-fold more IL-6^+^ B cells in Blk^+/−^.*lpr* mice than in B6.*lpr* mice (Figure 4D and E). Importantly, this same difference in IL-6^+^ B cell numbers was also noted in Blk^+/−^ mice in relation to B6 mice (Figure 4D and E). However, no differences in the numbers of IFNγ^+^ and IL-10^+^ B cells were observed among B6, Blk^+/−^, B6.*lpr*, and Blk^+/−^.*lpr* mice (data not shown). Interestingly, the IL-6^+^ B cells in all four genotypes were CD21^lo/−^ (Figure 4D), which is the same phenotype as the B cells that are the major source of IL-6 in mice with experimental autoimmune encephalomyelitis [45].

**Figure 6:**
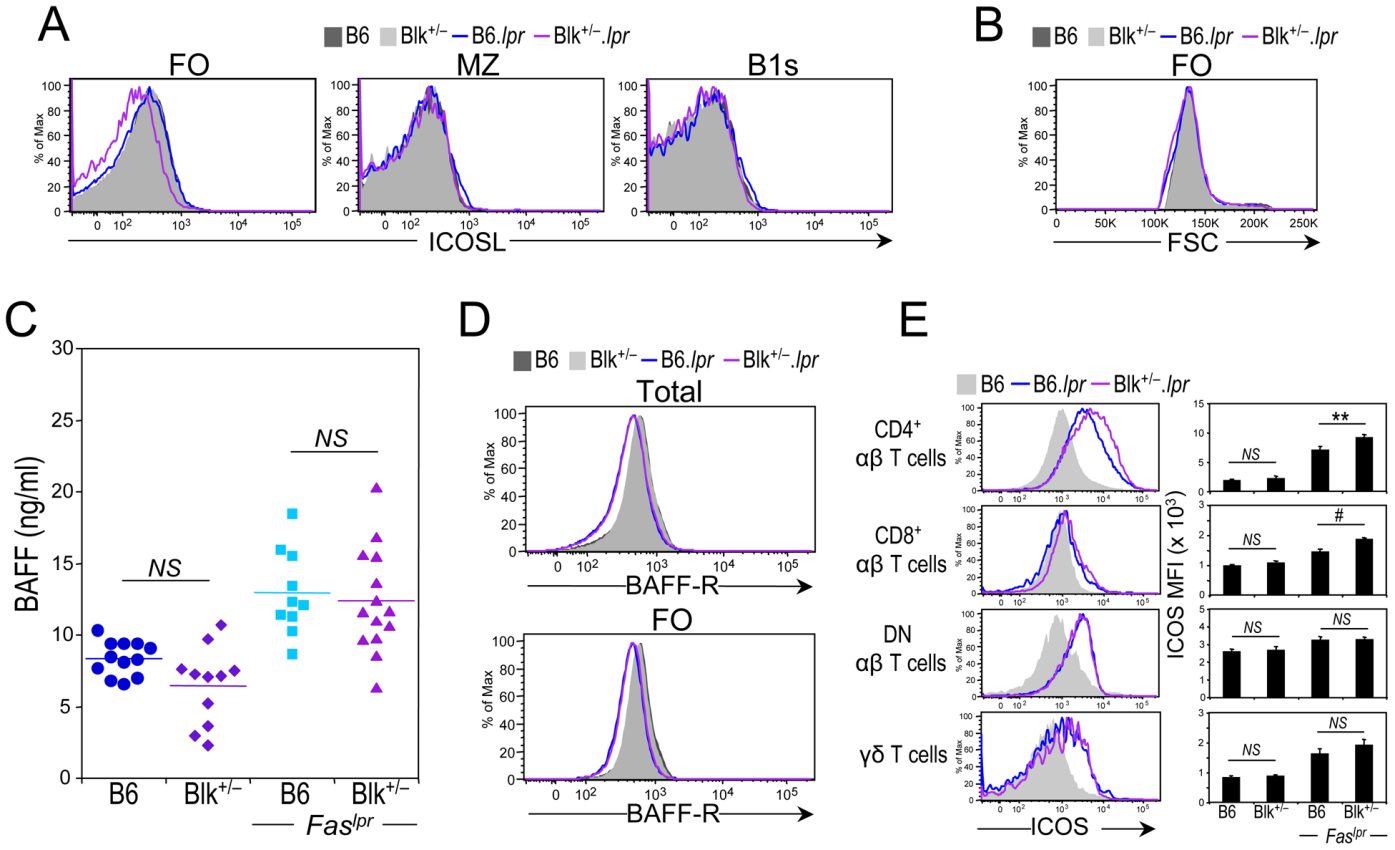
Evidence for augmented ICOS-ICOSL interactions in Blk^+/−^.*lpr* mice. (**A**) Representative histograms showing ICOSL levels on follicular (FO), marginal zone (MZ) and splenic B1 (B1s) B cells from 3-month-old B6 (n = 6), Blk^+/−^ (n = 6), B6.*lpr* (n = 7) and Blk^+/−^.*lpr* (n = 8) mice. (**B**) Histogram comparing B cell size, using FSC units, among 3-month-old B6 (n = 6), Blk^+/−^ (n = 6), B6.*lpr* (n = 7) and Blk^+/−^.*lpr* (n = 8) mice. (**C**) Comparison of the BAFF serum levels in 3-month-old B6, Blk^+/−^, B6.*lpr* and Blk^+/−^.*lpr* mice. Each symbol represents an individual mouse. (**D**) Representative histograms comparing BAFF-R levels on total (CD19^+^) (top) and FO B cells (bottom) from 3-month-old B6 (n = 8), Blk^+/−^ (n = 10), B6.*lpr* (n = 13) and Blk^+/−^.*lpr* (n = 12) mice. (**E**) Representative histograms showing the expression of ICOS on CD4^+^, CD8^+^, DN αβ, and γδ T cells subsets from the spleens of 3-month-old B6.*lpr* and Blk^+/−^.*lpr* mice. ICOS levels on the corresponding T cell subsets from age-matched B6 mice are also shown (shaded histogram). Adjacent graph compares ICOS levels (MFI) on each T cell subset between 3-month-old B6 (n = 6) and Blk^+/−^ (n = 6) mice and between 3-month-old B6.*lpr* (n = 7) and Blk^+/−^.*lpr* (n = 8) mice.

Regarding the ability of B cell subsets to function as APCs, we found that CD86 and IA^b^ surface levels were equivalent between B6.*lpr* and Blk^+/−^.*lpr* B cells (data not shown), but ICOSL surface levels on follicular (FO) B cells from Blk^+/−^.*lpr* mice were reduced compared to those on FO B cells from B6.*lpr* mice (Figure 6A). This phenomenon cannot be explained by an increase in the size of Blk^+/−^.*lpr* FO B cells, as their cell size was comparable to those of B6 and B6.*lpr* FO B cells (Figure 6B). Since BAFF-R signaling regulates ICOSL expression on B cells [46], [47], we next determined whether a decrease in BAFF serum levels, BAFF-R expression levels, or both can explain the lower ICOSL expression on Blk^+/−^.*lpr* FO B cells. Notably, while BAFF serum levels were higher, BAFF-R expression levels on B cells were lower, in both *Fas^lpr^* genotypes compared to both *Fas^wt^* genotypes (Figure 6C and D). Nonetheless, as no difference was observed between B6.*lpr* and Blk^+/−^.*lpr* mice in either their BAFF serum levels or their BAFF-R expression levels, it is unlikely that reduced BAFF-R signaling accounts for the lower ICOSL expression on Blk^+/−^.*lpr* FO B cells. It is possible, however, that the reduced ICOSL expression is a consequence of prior contact with ICOS^+^ T cells, as ICOSL expression on B cells is downregulated following engagement with ICOS on T cells [46], [48], [49]. To test this, we examined splenic T cell subsets for the expression of ICOS, a costimulatory molecule that is only expressed on activated T cells [50]. ICOS surface levels were higher on all T cell subsets from B6.*lpr* and Blk^+/−^.*lpr* mice than on those from B6 and Blk^+/−^ mice; however, the CD4^+^ and CD8^+^ T cell subsets from Blk^+/−^.*lpr* mice expressed significantly higher levels of ICOS than their B6.*lpr* counterparts (Figure 6E). These data suggest that the reduced ICOSL expression on Blk^+/−^.*lpr* FO B cells is due to augmented ICOS-ICOSL interactions in Blk^+/−^.*lpr* mice. Thus, B cells in 3-month-old Blk^+/−^.*lpr* mice have the capacity to play an autoantibody-independent role in the early stages of disease development, as evidenced by their enhanced ability to secrete IL-6 and to function as APCs.

### Blk is required to regulate T cell-mediated proinflammatory cytokine production

In light of the hyperactive B cell phenotype in 3-month-old Blk^+/−^.*lpr* mice, it was important to assess its effect on T cell numbers, phenotype and function. Interestingly, total T cell numbers were significantly higher in the Blk^+/−^.*lpr* spleen than in the B6.*lpr* spleen, with increased numbers of CD4^+^ and DN αβ T cells accounting for the higher T cell numbers (Table 1, Figure S4A and B). We also noted increased numbers of CD4^+^, CD8^+^, DN αβ, and γδ T cells in the Blk^+/−^ spleen relative to the B6 spleen (Table 1). However, despite the increase in total splenic T cell numbers in Blk^+/−^.*lpr* mice, the relative percentages of CD69^+^ cells within the αβ and γδ T cell subsets, as well as the relative percentages of CD4^+^ T cells with a memory cell phenotype (CD62L^l^°CD44^hi^), were comparable between the two *Fas^lpr^* genotypes (Figure S4C and D). Last, there was no difference in regulatory T cell numbers between B6 and Blk^+/−^ mice and between B6.*lpr* and Blk^+/−^.*lpr* mice (Table 1, Figure S4A).

**Figure 7:**
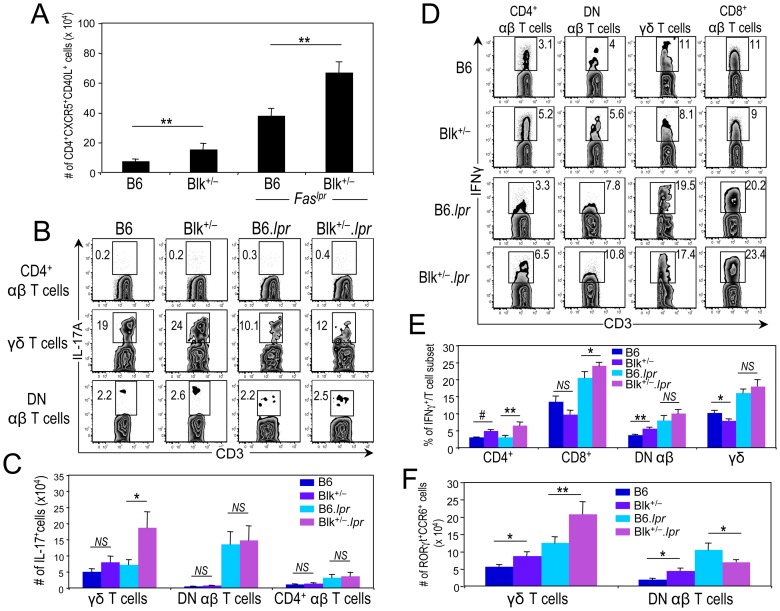
Reducing Blk expression in B6.*lpr* mice increases numbers of IFNγ-, IL-17A-, and IL-21-producing T cells. (**A**) Graph comparing the number of splenic Tfh cells, defined as CD4^+^ CXCR5^+^ CD40L^+^ cells, between 3-month-old B6 (n = 6) and Blk^+/−^ (n = 6) mice and between 3-month-old B6.*lpr* (n = 7) and Blk^+/−^.*lpr* (n = 8) mice. (**B**) pLN cells from 3-month-old B6, Blk^+/−^, B6.*lpr* and Blk^+/−^.*lpr* were stimulated with Leukocyte Activation Cocktail for 4 hours. Representative dot plots showing CD3 versus IL-17A expression in CD4^+^ αβ T cells (top), γδ T cells (middle) and in DN αβ T cells (bottom) from B6, Blk^+/−^, B6.*lpr* and Blk^+/−^.*lpr* mice. Numbers represent percentages of IL-17A^+^ cells. (**C**) Graph summarizing data in (**B**). Comparison of the number of IL-17A^+^ cells per T cell subset between 3-month-old B6 (n≥4) and Blk^+/−^ (n≥5) mice and between 3-month-old B6.*lpr* (n≥7) and Blk^+/−^.*lpr* (n≥8) mice. (**D**) Splenocytes from 3-month-old B6, Blk^+/−^, B6.*lpr* and Blk^+/−^.*lpr* mice were stimulated with Leukocyte Activation Cocktail for 4 hours. Representative dot plots show CD3 versus IFNγ expression in gated CD4^+^, CD8^+^, DN αβ and γδ T cell subsets. Numbers represent percentages of IFNγ^+^ cells. (**E**) Graph summarizing data in (**D**). Comparison of the percentage of IFNγ^+^ cells per T cell subset between 3-month-old B6 (n = 4) and Blk^+/−^ (n = 4) mice and between 3-month-old B6.*lpr* (n = 5) and Blk^+/−^.*lpr* (n = 5) mice. (**F**) Graph comparing the numbers of RORγt^+^ CCR6^+^ γδ T cells (γδ-17 cells) and DN αβ T cells (DN-17 cells) between the pLNs of 3-month-old B6 (n = 8) and Blk^+/−^ (n = 7) mice and between the pLNs of 3-month-old B6.*lpr* (n = 8) and Blk^+/−^.*lpr* (n = 12) mice. *p ≤ 0.05; **p ≤ 0.01; #*p* ≤ 0.001.

**Figure 8:**
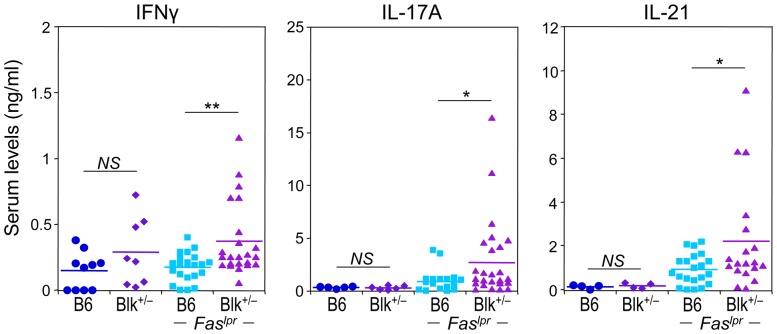
Reducing Blk expression in B6.*lpr* mice increases serum levels of IFNγ, IL-17A, and IL-21. Comparison of serum IFNγ, IL-17A, and IL-21 levels between 3-month-old B6 and Blk^+/−^ mice and between 3-month-old B6.*lpr* and Blk^+/−^.*lpr* mice. Each symbol represents an individual mouse. *p ≤ 0.05; **p ≤ 0.01.

Given that ICOS-ICOSL interactions appear to be augmented in Blk^+/−^.*lpr* mice and that ICOS-ICOSL signaling is associated with Th1, Tfh and Th17 effector fates [51]-[54], we next enumerated effector subsets and/or cytokine-producing cells in B6.*lpr* and Blk^+/−^.*lpr* mice. Significant increases in the numbers of Tfh cells as well as in the numbers of IFNγ- producing effector subsets were observed in Blk^+/−^.*lpr* mice relative to B6.*lpr* mice (Figure 7A, D, E). Furthermore, using both IL-17 production and phenotype (RORγt^+^ CCR6^+^) to identify γδ-17 cells, we detected significantly more γδ-17 cells in Blk^+/−^.*lpr* mice than in B6.*lpr* mice (Figure 7B, C, F). The numbers of IL-17^+^ CD4^+^ and DN αβ T cells, on the other hand, were equivalent between the two *Fas^lpr^* genotypes (Figure 7B, C, F). Lastly, consistent with increased local production of these proinflammatory cytokines, we found that the serum levels of IFNγ, IL-17A and IL-21 were significantly higher in Blk^+/−^.*lpr* mice than in B6.*lpr* mice (Figure 8). Together, these data demonstrate that reducing Blk expression in B6.*lpr* mice enhances proinflammatory cytokine production by both Blk-positive and -negative T cells.

**Figure 9:**
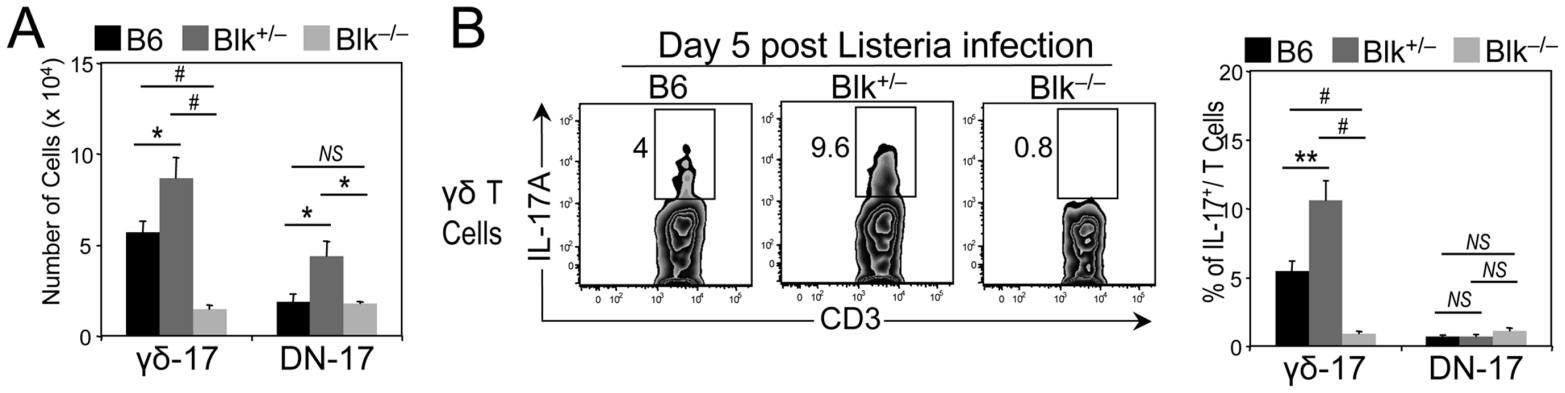
Different effects of Blk-haploinsufficiency and Blk-deficiency on *in vivo* γδ-17 effector function. (**A**) Graph comparing numbers of γδ-17 and DN-17 cells from B6 (n = 8), Blk^+/−^ (n = 7), and Blk^−/−^ (n = 6) mice. (**B**) B6, Blk^+/−^ and Blk^−/−^ mice were infected with *Listeria monocytogenes*. 5 days later, splenocytes were harvested and γδ-17 and DN-17 cells were *in vitro* stimulated for 4 hours with a cocktail of IL-23, IL-1, and Pam_3_Cys in the presence of brefeldin A. Dot plots showing CD3 versus IL-17A expression in gated γδ T cells from each of the three genotypes. Adjacent graph compares percentage of IL-17A^+^ per T cell subset in B6 (n = 10), Blk^+/−^ (n = 6), and Blk^−/−^ (n = 6) mice. *p ≤ 0.05; **p ≤ 0.01; #*p* ≤ 0.001.

We have previously shown that Blk-deficiency results in a selective loss of γδ-17 cells [23]. For this reason, we were intrigued to find that there were 50% more γδ-17 cells in Blk^+/−^ mice than in B6 mice (Figures 7F and 9A). As this number is quite different than the predicted one, based on a gene dose effect, of 50% fewer γδ-17 cells than B6 mice, we conclude that Blk-haploinsufficiency and Blk-deficiency differentially affect the development and/or homeostasis of γδ-17 cells. To determine whether Blk-haploinsufficiency and Blk-deficiency also have different effects on γδ-17 cell function, we compared the *in vivo* γδ-17 response in B6, Blk^+/−^ and Blk^−/−^ mice following infection with *Listeria monocytogenes*, a model pathogen known to elicit a robust γδ-17 response [55]. On day 5 post infection, we found that the percentage of IL-17^+^ γδ T cells is significantly higher in infected Blk^+/−^ mice than in infected B6 mice, while virtually no IL-17^+^ γδ T cells are detected in infected Blk^−/−^ mice (Figure 9B). Taken together, these data demonstrate that Blk-deficiency and Blk-haploinsufficiency have opposing effects on the generation and differentiation of γδ-17 cells.

### Blk is required to regulate macrophage-mediated IL-6 production

**Figure 10:**
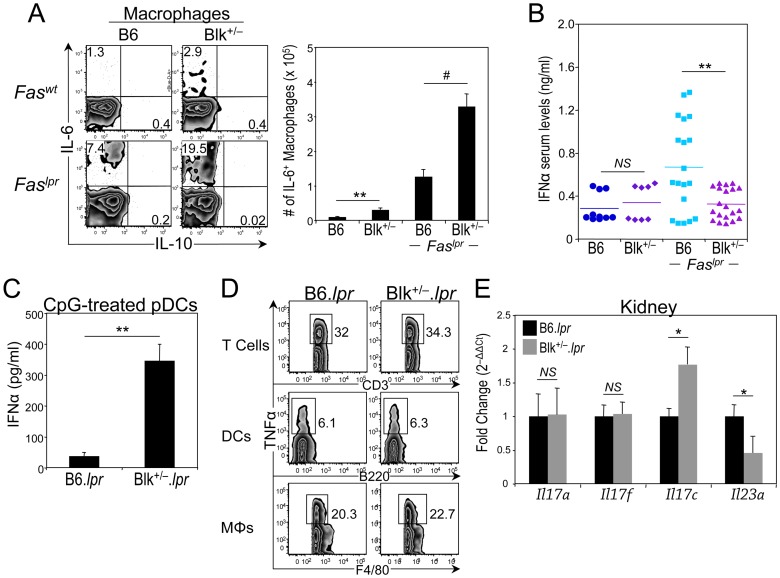
Reducing Blk expression in B6.*lpr* mice affects proinflammatory cytokine production by macrophages, dendritic cells and kidney. (**A**) Splenocytes from 3-month-old B6, Blk^+/−^, B6.*lpr* and Blk^+/−^.*lpr* mice were stimulated with Leukocyte Activation Cocktail for 4 hours. Dot plots showing IL-10 versus IL-6 expression in gated macrophages (F4/80^+^ CD3^–^ CD19^–^). Numbers represent percentage of IL-6^+^ and IL-10^+^ cells. Adjacent graph compares the number of IL-6^+^ macrophages between 3-month-old B6 (n = 4) and Blk^+/−^ (n = 4) mice and between 3-month-old B6.*lpr* (n = 7) and Blk^+/−^.*lpr* (n = 8) mice. (**B**) Comparison of serum IFNα levels between 3-month-old B6 and Blk^+/−^ mice and between 3-month-old B6.*lpr* and Blk^+/−^.*lpr* mice. Each symbol represents an individual mouse. (**C**) Purified pDCs from 2-month-old B6.*lpr* (n = 3) and Blk^+/−^.*lpr* (n = 3) mice were stimulated with CpG ODN 2395 for 24 hours and supernatants were collected and assayed for IFNα by ELISA. (**D**) Splenocytes from 3-month-old B6.*lpr* (n = 4) and Blk^+/−^.*lpr* mice (n = 4) were stimulated with Leukocyte Activation Cocktail for 4 hours. Dot plots showing CD3 versus TNFα expression in gated CD3^+^ T cells (top), B220 versus TNFα expression in gated DCs (center), and F4/80 versus TNFα expression in gated macrophages (MΦs) (bottom). Numbers represent percentages of TNFα^+^ cells. Very few (≤0.5%) B cells were TNFα^+^. (**E**) Expression data for *Il17a*, *Il17f*, *Il17c* and *Il23a* from RT^2^ profiler array. Data were normalized to *Gapdh* levels and are presented as fold change over 3-month-old B6.*lpr* kidney (set to 1). Data represent 4 to 5 mice per genotype. *p ≤ 0.05; **p ≤ 0.01; #*p* ≤ 0.001.

Notably, macrophages, in addition to B cells, produced elevated amounts of IL-6 in Blk^+/−^ and Blk^+/−^.*lpr* mice. Specifically, we observed a 3-fold increase in the number of IL-6^+^ macrophages in Blk^+/−^ mice compared to B6 mice and in Blk^+/−^.*lpr* mice compared to B6.*lpr* mice (Figure 10A). By contrast, nominal IL-6 production was detected in cDCs from all four genotypes (data not shown). Moreover, when we assayed for other proinflammatory cytokines, such as IL-1β, IL-12, IL-18, and IL-23, in the sera and the supernatants of short-term PMA/ionomycin-stimulated splenocytes from B6.*lpr* and Blk^+/−^.*lpr* mice, we were only able to detect IL-18, and it was produced in equivalent quantities in both *Fas^lpr^* genotypes (data not shown). These results demonstrate that reducing Blk expression in B6.*lpr* mice increases IL-6 production from macrophages, an immune cell that does not express Blk.

### Blk regulates pDC-mediated IFNα production

pDCs play an important role in autoimmunity through their production of type I interferon [32]. To determine whether their function is altered in Blk^+/−^.*lpr* mice, we measured serum levels of IFNα in B6.*lpr* and Blk^+/−^.*lpr* mice. Surprisingly, we observed significantly less IFNα in the sera of Blk^+/−^.*lpr* mice than in the sera of B6.*lpr* mice (Figure 10B). The decrease in serum IFNα levels is not the result of a defect in pDC development, as increased numbers of pDCs were detected in Blk^+/−^.*lpr* mice compared to B6.*lpr* mice (Table 1). However, the decrease in serum IFNα levels may be due to TLR tolerance, as a result of chronic TLR stimulation [56], since purified pDCs from younger Blk^+/−^.*lpr* mice were able to secrete IFNα, and at higher levels than pDCs from age-matched B6.*lpr* mice, following *in vitro* CpG stimulation (Figure 10C). Alternatively, the decreased serum levels of IFNα may be due to high local production of TNFα, which is known to inhibit INFα production from pDCs [57]. Although there were equivalent percentages of TNFα-producing T cells, DCs and macrophages following short-term PMA/ionomycin stimulation (Figure 10D), considerably more TNFα^+^ splenic T cells are present in Blk^+/−^.*lpr* mice than in B6.*lpr* mice, as a result of there being 40% more T cells in the Blk^+/−^.*lpr* spleen than in the B6.*lpr* spleen (Table 1). Collectively, these data suggest that the proinflammatory microenvironment established in the lymphoid tissue of Blk^+/−^.*lpr* mice negatively regulates IFNα secretion by pDCs.

### Blk is required to regulate proinflammatory cytokine production in the kidney

Because cytokines in the IL-23/IL-17 axis are critical in the pathogenesis of glomerulonephritis [35], [58], it is unclear why Blk^+/−^.*lpr* mice, which have elevated numbers of IL-17-producing T cells, do not develop this type of kidney disease. To address this, we compared the gene expression profiles of 3-month-old, pre-nephrotic B6.*lpr* and Blk^+/−^.*lpr* kidneys using the “Th17 for Autoimmunity and Inflammation” quantitative real-time PCR array. No differences were observed between the two genotypes in the expression of *Il17a* and *Il17f*, the IL-17 family members expressed by IL-17-producing T cells (Figure 10E). However, there was an ∼2-fold increase in *Il17c* expression in Blk^+/−^.*lpr* kidneys relative to B6.*lpr* kidneys (Figure 10E). IL-17C is an IL-17 family member that is expressed by epithelial cells [59], and it can play either a protective or pathogenic role, depending on the model system studied [60]. The only other gene whose expression differed significantly between the two genotypes was *Il23a*, in which we noted an ∼2-fold decrease in expression in Blk^+/−^.*lpr* kidneys compared to B6.*lpr* kidneys (Figure 10E, data not shown). As IL-23 signaling is required for the expansion and differentiation of unconventional IL-17-producing T cell effectors [61], which in turn drive the development of glomerulonephritis in B6.*lpr* mice [35], these findings suggest that a reduction in IL-23 production in the kidney alters disease pathogenesis in Blk^+/−^.*lpr* mice.

## Discussion

With the completion of both the Human Genome Project and the International HapMap Project, researchers are able to scan markers across the human genome to identify genes that contribute to complex human diseases, such as SLE [62]. One of the newly identified SLE susceptibility genes is *BLK* [4]–[10], and SNPs that map to this locus result in a reduction in *BLK* expression [5], [11]–[13]. In this report, we follow-up on the identification of *BLK* as a susceptibility gene by determining whether and how reduced *BLK* expression contributes to SLE disease development. Using the autoimmune-prone B6.*lpr* mouse model, in which we solely reduced *Blk* expression levels, we noted enhanced proinflammatory cytokine production by both Blk-positive and -negative immune cells in addition to early onset lymphoproliferation, proteinuria, and kidney disease. Together, these findings not only confirm *BLK* as a *bona fide* susceptibility gene for SLE but also reveal new functions for Blk in immune cell activation and regulation.

Systemic autoimmune disease is the result of synergistic actions of multiple susceptibility genes, with each susceptibility gene making a small contribution to disease development [63]. By analyzing both Blk^+/−^ and Blk^+/−^.*lpr* mice, we are unraveling how reduced Blk expression alters immune cell activation and in turn contributes to the development of autoimmunity. The increased production of IL-6 by B cells and macrophages in 3-month-old Blk^+/−^ mice may foster a proinflammatory microenvironment that supports the development and/or maintenance of T cell subsets that have the potential to produce IFNγ, IL-17A, IL-21 and TNFα. Indeed, IL-6 has been shown to play a role in the generation of these cytokine-producing T cells [64]–[66]. Not until the *Fas^lpr^* mutation is introduced onto the Blk^+/−^ background, however, do these T cell subsets become activated and secrete their respective cytokines. Enhanced ICOS-ICOSL signaling in the Blk^+/−^.*lpr* mouse model may contribute to their differentiation into cytokine-producing effectors at 3 months of age. In Blk^+/−^.*lpr* mice, the reduced ICOSL expression on FO B cells combined with the increased ICOS expression on CD4^+^ and CD8^+^ T cells strongly suggest prior and augmented ICOS-ICOSL signaling [46], [48], [49]. Interestingly, similar changes in ICOS and ICOSL expression are also observed in SLE patients [49] and in NZB/W F_1_ mice, a lupus mouse model in which ICOS/ICOSL interactions are known to contribute to disease development [46]. Importantly, the interactions between B cells and T cells are reciprocal at this stage, as evidenced by increases in plasma cell numbers and serum IgG levels in Blk^+/−^.*lpr* mice compared to B6.*lpr* mice. Eventually, the increased production of TNFα, along with other proinflammatory cytokines, culminates in accelerated onset of proteinuria in 60% of 5-month-old Blk^+/−^.*lpr* mice. This high degree of penetrance in our experimental model is striking given that a similar incidence of proteinuria is observed in MRL.*lpr* mice at 3 to 4 months of age [67] and in NZB/W F_1_ mice at 6 to 7 months of age [46].

The vast majority of allelic polymorphisms, on which gene expression studies have been performed, result in a change in protein expression or activity, not a null mutation [5], [11], [12], [68]. Consequently, to study how these allelic polymorphisms increase disease risk, it is critical to use animal models in which gene expression approximates that of the human risk allele(s). This is especially true for *BLK*, as we have discovered a major phenotypic difference between Blk^+/−^ and Blk^−/−^ mice, which could have an effect on disease progression and pathogenesis. Even though both γδ-17 and DN-17 cells express Blk, Blk^−/−^ mice exhibit a ∼75% decrease in γδ-17 cell numbers, but no change in DN-17 cell numbers, compared to B6 mice. Surprisingly, instead of observing a phenotype consistent with a gene dosage effect, Blk^+/−^ mice have ∼50% more γδ-17 cells and ∼100% more DN-17 cells than B6 mice. Moreover, following infection with *Listeria monocytogenes*, which elicits both γδ-17 and DN-17 cell responses [61], we found that the percentage of IL-17^+^ γδ T cells, but not of IL-17^+^ DN αβ T cells, was significantly greater in infected Blk^+/−^ mice than in infected B6 mice. By contrast, virtually no IL-17^+^ γδ T cells were detected in the spleen of infected Blk^−/−^ mice. Notably, there is a similar bias towards γδ-17 cells in Blk^+/−^.*lpr* mice, as there are 3-fold more CCR6^+^ RORγt^+^ γδ T cells than CCR6^+^ RORγt^+^ DN αβ T cells in Blk^+/−^.*lpr* mice yet equivalent numbers of these cells in B6.*lpr* mice. IL-17A has the attributes of a key effector cytokine in the Blk^+/−^.*lpr* model, not only because its serum levels are significantly higher in Blk^+/−^.*lpr* mice than in B6.*lpr* mice, but also because it is the only T cell cytokine whose serum levels correlate positively with IgG serum levels in Blk^+/−^.*lpr* mice (r^2^  =  0.551; *p*  =  0.04). This correlation suggests that IL-17A mediates immunoglobulin class switch and antibody production in our experimental mouse model, which is in agreement with other reports demonstrating a role for IL-17A in IgG production [58], [69]-[71]. Accordingly, if our study of the role of *BLK* in the development of SLE had used Blk^−/−^.*lpr* mice, then there would be no functional γδ-17 cells and, most likely, no enhanced IL-17A and IgG production at 3 months of age. Importantly, other phenotypes that are observed in 3- and 5-month-old Blk^+/−^.*lpr* mice, as a direct or indirect consequence of elevated IL-17A production, may be delayed or may not even occur in Blk^−/−^.*lpr* mice.

There are three key steps in the development of systemic autoimmunity, commencing with loss of tolerance to self antigens, progressing to dysregulation of both the innate and adaptive immune systems, and ending with inflammation and tissue damage [1], [72], [73]. Previous studies, using different mouse models of lupus, have identified susceptibility genes and gene clusters that act at each of the three steps [1], [72], [73]. Since B cell tolerance is maintained in Blk^+/−^ 3H9 Tg mice role, we conclude that *BLK* variants do not confer susceptibility to SLE by breaking B cell tolerance to self-antigens and, therefore, do not act at the first step of this model. Our findings, instead, reveal a role for Blk in the regulation of a proinflammatory cytokine network, involving cells of both the innate and adaptive immune systems. Such a regulatory function would indicate that *BLK* variants act at the second step in the three-step model. Notably, other genes and gene clusters that are also reported to act at the second step and participate in the dysregulation of innate and adaptive immune cell function include *Fas*, *Lyn*, *Sle2*, *Sle3*, *Tlr7*, and *Ptpn6* [1], [72], [73].

Since the discovery of *BLK* as a SLE susceptibility gene, SNPs in the *BLK* locus have also been shown to associate with disease risk in systemic sclerosis [74], rheumatoid arthritis [75], [76], Sjögren's syndrome [77], and Kawasaki disease [78], [79]. The fact that *BLK* risk alleles are shared by multiple autoimmune diseases suggests that the risk alleles promote disease through a common underlying mechanism. We have demonstrated enhanced production of the proinflammatory cytokines IL-6, TNFα, IFNγ, IL-17A and IL-21 in Blk^+/−^.*lpr* mice compared to age-matched B6.*lpr* mice. Given that each one of these cytokines plays an essential role in disease development and/or pathogenesis in mouse models of these autoimmune diseases [35], [80]–[88] and that elevated plasma levels of IL-6, TNFα, IL-17A and IL-21 are detected in patients with SLE [89]–[91], systemic sclerosis [90], [92], [93], rheumatoid arthritis [90], [94]–[96], Sjögren's syndrome [97]–[99], and Kawasaki disease [100]–[102], we propose that dysregulation of a proinflammatory cytokine network is the common mechanism by which *BLK* risk alleles promote autoimmune disease development.

In summary, we have demonstrated that solely reducing Blk expression levels in autoimmune-prone B6.*lpr* mice results in elevated proinflammatory cytokine production prior to the onset of proteinuria and nephrosis. Accordingly, we conclude that SNPs in the *BLK* locus increase risk to SLE, and to other autoimmune diseases, through the dysregulation of a proinflammatory cytokine network. Determining the hierarchy and interdependence of the cytokines in this network may lead to new tests for early detection as well as to new therapies that can be implemented in at risk individuals prior to the onset of autoimmune disease.

## Supporting Information

Figure S1 **Comparison of autoimmune phenotypes between 3-month-old and 5-month-old B6.** ***lpr*** **and Blk^+/−^.** ***lpr*** **mice.** (**A**) Comparison of serum ANA levels between 5-month-old B6.*lpr* and Blk^+/−^.*lpr* mice. Each symbol represents an individual mouse. (**B**) Comparison of the cellularity of the spleen and pLNs between 3-month-old B6.*lpr* and Blk^+/−^.*lpr* mice. Each symbol represents an individual mouse. (**C**) Comparison of serum ANA levels between 3-month-old B6.*lpr* and Blk^+/−^.*lpr* mice. Shaded gray band represents range of ANA serum levels in age-matched B6 and Blk^+/−^ mice. Each symbol represents an individual mouse.
(DOCX)

Figure S2 **Enlarged electron micrographs of glomeruli from 5-month-old B6.** ***lpr*** **and Blk^+/−^.** ***lpr*** **mice.** The capillary lumen (denoted as CL) in the B6.*lpr* glomerulus (left panel) is open and red blood cells are visible within the lumen. By contrast, the capillary lumen in the Blk^+/−^.*lpr* glomerulus is dramatically narrowed. Rectangular boxes in both panels highlight normal (left panel) and shortened/fused (right panel) podocyte foot processes. Line in bottom of micrographs represents 2 μm.
(DOCX)

Figure S3 **Effect of reducing Blk expression levels on B cell development in B6.** ***lpr*** **mice.** (**A**) Far left panel: Dot plots showing CD19 versus CD93 expression on total splenocytes from 3-month-old B6 (n = 19), Blk^+/−^ (n = 16), B6.*lpr* (n = 23) and Blk^+/−^.*lpr* (n = 27) mice. Numbers in plots represent percentages of transitional (CD19^+^ CD93^+^) and mature (CD19^+^ CD93^−^) B cells. Left center panel: Dot plots showing CD21 versus CD23 expression on gated mature B cells. Numbers in plots represent percentages of FO B cells (CD23^hi^ CD21^lo^), MZ B cells (CD23^l^°CD21^hi^), and pre-plasmablasts (CD23^l^°CD21^lo^). Right two panels: Dot plots showing IgM versus CD5 expression on lymphocytes in the spleen and peritoneal cavity (PEC). Numbers in plots represent percentages of B1 B cells (CD5^lo^ IgM^+^). (**B**) Graphs comparing the percentages of MZ B cells, splenic B1 (B1s) B cells, and pre-plasmablasts (pre-PB) between 3-month-old B6 and Blk^+/−^ mice and between 3-month-old B6.*lpr* and Blk^+/−^.*lpr* mice.
(DOCX)

Figure S4 **Effect of reducing Blk expression levels on T cell development in B6.** ***lpr*** **mice.** (**A**) Far left panel: Dot plots showing CD3 versus TCRβ expression on total splenocytes from 3-month-old B6 (n = 19), Blk^+/−^ (n = 16), B6.*lpr* (n = 23) and Blk^+/−^.*lpr* (n = 27) mice. Numbers in plots represent percentages of αβ T cells. Left center panel: Dot plots showing CD8 versus CD4 expression on gated αβ T cells. Numbers represent percentages of cells in three of the quadrants. Center panel: Histograms showing B220 expression on gated DN αβ T cells. Numbers in histograms represent percentage of B220^+^ DN αβ T cells. Right center panel: Dot plots showing CD3 versus TCRγδ expression on total splenocytes. Numbers in plots represent percentages of γδ T cells. Far right panel: Dot plots showing CD25 versus Foxp3 expression in gated CD4^+^ αβ T cells. Numbers in plots represent percentages of regulatory T cells. (**B**) Graph comparing the percentages of different T cell subsets between 3-month-old B6 and Blk^+/−^ mice and between 3-month-old B6.*lpr* and Blk^+/−^.*lpr* mice. *p≤0.05; **p≤0.01. (**C**) Histograms comparing CD69 expression on gated splenic CD4^+^, CD8^+^, DN αβ, and γδ T cell subsets from 3-month-old B6.*lpr* and Blk^+/−^.*lpr* mice. CD69 expression levels on the corresponding splenic T cell subsets from age-matched B6 mice are also shown (shaded histogram). (**D**) Dot plots showing CD44 versus CD62L expression on gated CD4^+^ splenocytes from 3-month-old B6, Blk^+/−^, B6.*lpr* and Blk^+/−^.*lpr* mice. Numbers in plots represent percentages of naive (CD62L^hi^ CD44^lo^), effector (CD62L^hi^ CD44^hi^), and memory (CD62L^l^°CD44^hi^) CD4^+^ T cells.
(DOCX)
