# Supplementary material for: Reduced B Lymphoid Kinase (Blk) Expression Enhances Proinflammatory Cytokine Production and Induces Nephrosis in C57BL/6-lpr/lpr Mice
Source: PLoS One. 2014 Mar 17;9(3):e92054. doi: 10.1371/journal.pone.0092054 (PMC3956874; doi:10.1371/journal.pone.0092054)
Supplement: Figure S1 — Comparison of autoimmune phenotypes between 3-month-old and 5-month-old B6. lpr and Blk+/−. lpr mice. (A) Comparison of serum ANA levels between 5-month-old B6.lpr and Blk+/−.lpr mice. Each symbol represents an individual mouse. (B) Comparison of the cellularity of the spleen and pLNs between 3-month-old B6.lpr and Blk+/−.lpr mice. Each symbol represents an individual mouse. (C) Comparison of serum ANA levels between 3-month-old B6.lpr and Blk+/−.lpr mice. Shaded gray band represents range of ANA serum levels in age-matched B6 and Blk+/− mice. Each symbol represents an individual mouse. (DOCX) [file pone.0092054.s001.docx]

**Figure S1. Comparison of autoimmune phenotypes between 3-month-old and 5-month-old B6.*lpr* and Blk^+/−^.*lpr* mice.** (**A**) Comparison of serum ANA levels between 5-month-old B6.*lpr* and Blk^+/−^.*lpr* mice. Each symbol represents an individual mouse. (**B**) Comparison of the cellularity of the spleen and pLNs between 3-month-old B6.*lpr* and Blk^+/−^.*lpr* mice. Each symbol represents an individual mouse. (**C**) Comparison of serum ANA levels between 3-month-old B6.*lpr* and Blk^+/−^.*lpr* mice. Shaded gray band represents range of ANA serum levels in age-matched B6 and Blk^+/−^ mice. Each symbol represents an individual mouse.
