# Supplementary material for: Reduced B Lymphoid Kinase (Blk) Expression Enhances Proinflammatory Cytokine Production and Induces Nephrosis in C57BL/6-lpr/lpr Mice
Source: PLoS One. 2014 Mar 17;9(3):e92054. doi: 10.1371/journal.pone.0092054 (PMC3956874; doi:10.1371/journal.pone.0092054)
Supplement: Figure S2 — Enlarged electron micrographs of glomeruli from 5-month-old B6. lpr and Blk+/−. lpr mice. The capillary lumen (denoted as CL) in the B6.lpr glomerulus (left panel) is open and red blood cells are visible within the lumen. By contrast, the capillary lumen in the Blk+/−.lpr glomerulus is dramatically narrowed. Rectangular boxes in both panels highlight normal (left panel) and shortened/fused (right panel) podocyte foot processes. Line in bottom of micrographs represents 2 μm. (DOCX) [file pone.0092054.s002.docx]

Kidney


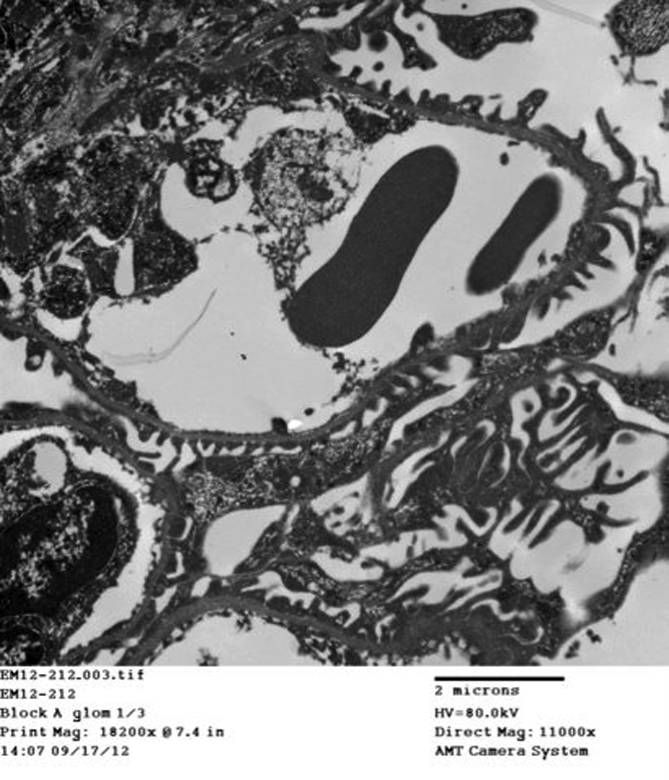


CL

B6.*lpr*


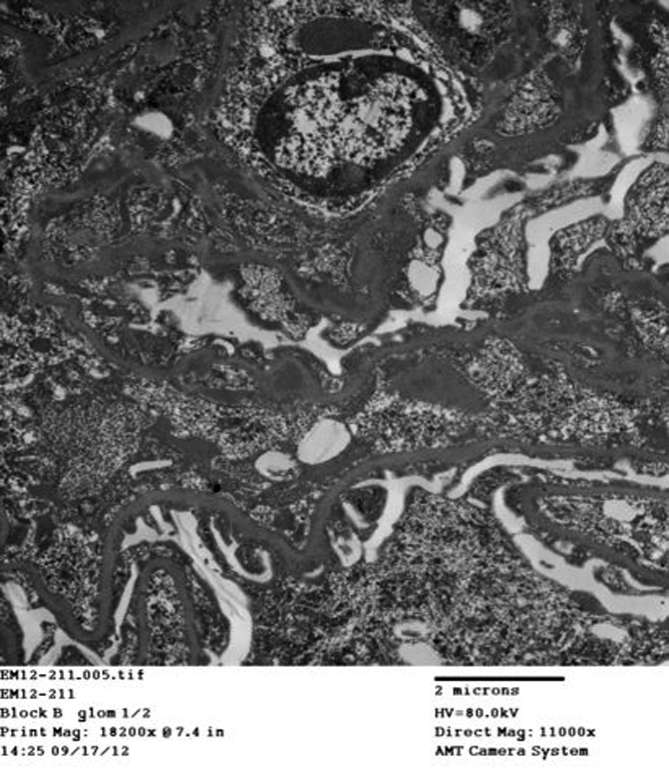


CL

Blk^+/–^.*lpr*

**Figure S2. Enlarged electron micrographs of glomeruli from 5-month-old B6.*lpr* and Blk^+/−^.*lpr* mice.** The capillary lumen (denoted as CL) in the B6.*lpr* glomerulus (left panel) is open and red blood cells are visible within the lumen., By contrast, the capillary lumen in the Blk^+/−^.*lpr* glomerulus is dramatically narrowed. Rectangular boxes in both panels highlight normal (left panel) and shortened/fused (right panel) podocyte foot processes. Line in bottom of micrographs represents 2 µm.
