# Supplementary material for: Reduced B Lymphoid Kinase (Blk) Expression Enhances Proinflammatory Cytokine Production and Induces Nephrosis in C57BL/6-lpr/lpr Mice
Source: PLoS One. 2014 Mar 17;9(3):e92054. doi: 10.1371/journal.pone.0092054 (PMC3956874; doi:10.1371/journal.pone.0092054)
Supplement: Figure S3 — Effect of reducing Blk expression levels on B cell development in B6. lpr mice. (A) Far left panel: Dot plots showing CD19 versus CD93 expression on total splenocytes from 3-month-old B6 (n = 19), Blk+/− (n = 16), B6.lpr (n = 23) and Blk+/−.lpr (n = 27) mice. Numbers in plots represent percentages of transitional (CD19+ CD93+) and mature (CD19+ CD93−) B cells. Left center panel: Dot plots showing CD21 versus CD23 expression on gated mature B cells. Numbers in plots represent percentages of FO B cells (CD23hi CD21lo), MZ B cells (CD23l°CD21hi), and pre-plasmablasts (CD23l°CD21lo). Right two panels: Dot plots showing IgM versus CD5 expression on lymphocytes in the spleen and peritoneal cavity (PEC). Numbers in plots represent percentages of B1 B cells (CD5lo IgM+). (B) Graphs comparing the percentages of MZ B cells, splenic B1 (B1s) B cells, and pre-plasmablasts (pre-PB) between 3-month-old B6 and Blk+/− mice and between 3-month-old B6.lpr and Blk+/−.lpr mice. (DOCX) [file pone.0092054.s003.docx]

**Figure S3. Effect of reducing Blk expression levels on B cell development in B6.*lpr* mice.** (**A**) Far left panel: Dot plots showing CD19 versus CD93 expression on total splenocytes from 3-month-old B6, Blk^+/−^, B6.*lpr* and Blk^+/−^.*lpr* mice. Numbers in plots represent percentages of transitional (CD19^+^ CD93^+^) and mature (CD19^+^ CD93^−^) B cells. Left center panel: Dot plots showing CD21 versus CD23 expression on gated mature B cells. Numbers in plots represent percentages of FO B cells (CD23^hi^ CD21^lo^), MZ B cells (CD23^lo^ CD21^hi^), and pre-plasmablasts (CD23^lo^ CD21^lo^). Right two panels: Dot plots showing IgM versus CD5 expression on lymphocytes in the spleen and peritoneal cavity (PEC). Numbers in plots represent percentages of B1 B cells (CD5^lo^ IgM^+^). (**B**) Graphs comparing the percentages of MZ B cells, splenic B1 (B1s) B cells, and pre-plasmablasts (pre-PB) between 3-month-old B6 (n=19) and Blk^+/−^ (n=16) mice and between 3-month-old B6.*lpr* (n=23) and Blk^+/−^.*lpr* (n=27) mice.
