# Supplementary material for: Reduced B Lymphoid Kinase (Blk) Expression Enhances Proinflammatory Cytokine Production and Induces Nephrosis in C57BL/6-lpr/lpr Mice
Source: PLoS One. 2014 Mar 17;9(3):e92054. doi: 10.1371/journal.pone.0092054 (PMC3956874; doi:10.1371/journal.pone.0092054)
Supplement: Figure S4 — Effect of reducing Blk expression levels on T cell development in B6. lpr mice. (A) Far left panel: Dot plots showing CD3 versus TCRβ expression on total splenocytes from 3-month-old B6 (n = 19), Blk+/− (n = 16), B6.lpr (n = 23) and Blk+/−.lpr (n = 27) mice. Numbers in plots represent percentages of αβ T cells. Left center panel: Dot plots showing CD8 versus CD4 expression on gated αβ T cells. Numbers represent percentages of cells in three of the quadrants. Center panel: Histograms showing B220 expression on gated DN αβ T cells. Numbers in histograms represent percentage of B220+ DN αβ T cells. Right center panel: Dot plots showing CD3 versus TCRγδ expression on total splenocytes. Numbers in plots represent percentages of γδ T cells. Far right panel: Dot plots showing CD25 versus Foxp3 expression in gated CD4+ αβ T cells. Numbers in plots represent percentages of regulatory T cells. (B) Graph comparing the percentages of different T cell subsets between 3-month-old B6 and Blk+/− mice and between 3-month-old B6.lpr and Blk+/−.lpr mice. *p≤0.05; **p≤0.01. (C) Histograms comparing CD69 expression on gated splenic CD4+, CD8+, DN αβ, and γδ T cell subsets from 3-month-old B6.lpr and Blk+/−.lpr mice. CD69 expression levels on the corresponding splenic T cell subsets from age-matched B6 mice are also shown (shaded histogram). (D) Dot plots showing CD44 versus CD62L expression on gated CD4+ splenocytes from 3-month-old B6, Blk+/−, B6.lpr and Blk+/−.lpr mice. Numbers in plots represent percentages of naive (CD62Lhi CD44lo), effector (CD62Lhi CD44hi), and memory (CD62Ll°CD44hi) CD4+ T cells. (DOCX) [file pone.0092054.s004.docx]

**Figure S4. Effect of reducing Blk expression levels on T cell development in B6.*lpr* mice.** (**A**) Far left panel: Dot plots showing CD3 versus TCRβ expression on total splenocytes from 3-month-old B6, Blk^+/−^, B6.*lpr* and Blk^+/−^.*lpr* mice. Numbers in plots represent percentages of αβ T cells. Left center panel: Dot plots showing CD8 versus CD4 expression on gated αβ T cells. Numbers represent percentages of cells in three of the quadrants. Center panel: Histograms showing B220 expression on gated DN αβ T cells. Numbers in histograms represent percentage of B220^+^ DN αβ T cells. Right center panel: Dot plots showing CD3 versus TCRγδ expression on total splenocytes. Numbers in plots represent percentages of γδ T cells. Far right panel: Dot plots showing CD25 versus Foxp3 expression in gated CD4^+^ αβ T cells. Numbers in plots represent percentages of regulatory T cells. (**B**) Graph comparing the percentages of different T cell subsets between 3-month-old B6 (n=19) and Blk^+/−^ (n=16) mice and between 3-month-old B6.*lpr* (n=23) and Blk^+/−^.*lpr* (n=27) mice. *p≤0.05; **p≤0.01. (**C**) Histograms comparing CD69 expression on gated splenic CD4^+^, CD8^+^, DN αβ, and γδ T cell subsets from 3-month-old B6.*lpr* and Blk^+/−^.*lpr* mice. CD69 expression levels on the corresponding splenic T cell subsets from age-matched B6 mice are also shown (shaded histogram). (**D**) Dot plots showing CD44 versus CD62L expression on gated CD4^+^ splenocytes from 3-month-old B6, Blk^+/−^, B6.*lpr* and Blk^+/−^.*lpr* mice. Numbers in plots represent percentages of naive (CD62L^hi^ CD44^lo^), effector (CD62L^hi^ CD44^hi^), and memory (CD62L^lo^ CD44^hi^) CD4^+^ T cells.
